# Lipidomics of Environmental Microbial Communities. I: Visualization of Component Distributions Using Untargeted Analysis of High-Resolution Mass Spectrometry Data

**DOI:** 10.3389/fmicb.2021.659302

**Published:** 2021-07-23

**Authors:** Nicole J. Bale, Su Ding, Ellen C. Hopmans, Milou G. I. Arts, Laura Villanueva, Christine Boschman, Andreas F. Haas, Stefan Schouten, Jaap S. Sinninghe Damsté

**Affiliations:** ^1^Department of Marine Microbiology and Biogeochemistry, Royal Netherlands Institute for Sea Research, Texel, Netherlands; ^2^Department of Earth Sciences, Faculty of Geosciences, Utrecht University, Utrecht, Netherlands

**Keywords:** lipids, liquid chromatography mass spectrometry, lipidome, lipidomics, MZmine, Black Sea

## Abstract

Lipids, as one of the main building blocks of cells, can provide valuable information on microorganisms in the environment. Traditionally, gas or liquid chromatography coupled to mass spectrometry (MS) has been used to analyze environmental lipids. The resulting spectra were then processed through individual peak identification and comparison with previously published mass spectra. Here, we present an untargeted analysis of MS^1^ spectral data generated by ultra-high-pressure liquid chromatography coupled with high-resolution mass spectrometry of environmental microbial communities. Rather than attempting to relate each mass spectrum to a specific compound, we have treated each mass spectrum as a component, which can be clustered together with other components based on similarity in their abundance depth profiles through the water column. We present this untargeted data visualization method on lipids of suspended particles from the water column of the Black Sea, which included >14,000 components. These components form clusters that correspond with distinct microbial communities driven by the highly stratified water column. The clusters include both known and unknown compounds, predominantly lipids, demonstrating the value of this rapid approach to visualize component distributions and identify novel lipid biomarkers.

## Introduction

Lipids are the main building blocks of microorganisms and occur ubiquitously in the environment. A large number of lipids are synthesized by many different genera and orders of microbes but some lipids are unique to specific organisms or groups of organisms or to specific biogeochemical processes (e.g., Koga et al., 1998; Cvejic et al., 2000; Sinninghe Damsté et al., 2002b; Belt et al., 2007; Rossel et al., 2008; Bauersachs et al., 2009; Elling et al., 2017; Rush and Sinninghe Damsté, 2017; Longo et al., 2018; Gutiérrez et al., 2020), and hence serve as biomarker lipids. Furthermore, intact polar lipids (IPLs) are thought to degrade rapidly upon cell death indicating recent activity of microbial cells (White et al., 1979; Harvey et al., 1986). Therefore, analysis of IPLs and other lipids can, complementary to microbiological and molecular methods, provide valuable information on the diversity and activity of microorganisms in the environment. Early biomarker lipid studies of the microbial composition in the environment utilized gas chromatography (GC) often coupled to mass spectrometry (MS) to detect lipid biomarker such as steroids (Gaskell and Eglinton, 1976; Summons et al., 1992), and in particular phospholipid derived fatty acids (PLFAs) (White et al., 1979). In recent decades, the range of biomarker lipids utilized in environmental studies has been extended to include direct analysis of larger IPLs using liquid chromatography (LC) coupled to MS (e.g., Sturt et al., 2004).

Traditionally, the range of IPLs detected in the environment using LC-MS are constrained through individual peak identification and comparison with previously published mass spectra or standards (Sturt et al., 2004; Ertefai et al., 2008; Lipp and Hinrichs, 2009; Schubotz et al., 2009, 2018; Van Mooy and Fredricks, 2010; Popendorf et al., 2011; Brandsma et al., 2012; Gibson et al., 2013; Bale et al., 2016; Ding et al., 2020). This generally results in the detection only of dominant (groups of) known IPLs. With the advent of ultra-high-pressure liquid chromatography coupled with multistage high-resolution mass spectrometry (UHPLC-HRMS^*n*^) the richness of the mass spectral data has significantly increased, leading to the identification of many new lipid compounds (e.g., Liu et al., 2012a,b; Moore et al., 2013, 2016; Zhu et al., 2014a,b; Yoshinaga et al., 2015; Sollai et al., 2018; Bale et al., 2019b), but this identification is selective and tedious and could be sped up by using computational approaches. Furthermore, compounds with unknown mass spectra may be ignored even though they could be indicative of the abundance of specific microbes.

Recently, a number of studies have applied lipidomic data handling strategies in marine environments (Hunter et al., 2015; Collins et al., 2016; Cutignano et al., 2016; Becker et al., 2018a; Li et al., 2018; Saleem et al., 2019; Zeng et al., 2019). These studies describe various methods for lipidomic workflows such as high-throughput annotation and identification of lipids in HRMS data and statistical analysis of the outputted lipid data. Some of which have taken an untargeted approach, rather than attempting to relate each mass spectrum to a certain compound, they extract all spectral components (sometimes denoted as “features”) (Pluskal et al., 2010, 2020). The composition of spectral components can then be used to, e.g., define specific environmental niches.

Here, we perform an untargeted analysis of the lipidome of the Black Sea. We extract components based on high resolution MS^1^ spectra from UHPLC-HRMS analysis of lipid extracts of suspended particles and use a statistical approach to compare the individual abundance depth profiles of all components. These depth profiles are clustered by their similarity to one another, without bias toward known or abundant components. We compare these findings with previous studies, which have examined the lipidome of the Black Sea water column using traditional IPL identification (e.g., Neretin et al., 2007; Wakeham et al., 2007; Schubotz et al., 2009; Sollai et al., 2018). Our rapid visualization method lays a foundation for the study of Ding et al. (2021), reported in parallel to this study, which used information theory framework combined with molecular networking to investigate lipid diversity and specificity as well as identify novel lipids.

## Materials and Methods

### Sampling and Environmental Setting

Suspended particulate matter (SPM) at various water depth in the water column (Table 1) was collected during two cruises in 2013 and 2017 in the Black Sea. The 2013 Black Sea SPM was collected from 50 to 2000 meter below sea level (mbsl; see Table 1 for depths) during the PHOXY cruise (June 2013) aboard of the *R/V Pelagia* (Kraal et al., 2017; Sollai et al., 2018). The sampling station (PHOX2) was located at 42°53.8′N, 30°40.7′E in the western gyre of the Black Sea. The 2017 Black Sea SPM (50–2000 m, Table 1) was collected during the 64PE418 cruise (March 2017) also aboard of the *R/V Pelagia*. For the latter, the sampling station (Station “4”) was located at 42°46.9′N, 29°21.1′E. Because the two SPM profiles (2013 and 2017) were collected from different stations (∼60 nautical miles apart, although both in the center of the western gyre of the Black Sea), they represent two distinct sample sets, both temporally and spatially.

**TABLE 1:** Depth of SPM sampling and hydrological properties in the water column in the center of the western gyre of the Black Sea at sampling station PHOX2 in March 2013 and station 64PE418-4 in June 2017.

**Year**	**Redox zone**	**Depth (mbsl)**	**O_2_ (μ mol kg^–1^)**	**HS^–^ (μ M)**	**NO_3_^–^ (μ mol L^–1^)**	**Salinity**	**Density**
**2013**	Oxic	50	121	<DL	1.3	19.4	14.9
		70	2.2	<DL	2.5	20.1	15.7
	Suboxic	80	<DL	<DL	2.5	20.4	15.9
		85	<DL	<DL	0.3	20.5	16.0
		90	<DL	<DL	0.04	20.6	16.0
		95	<DL	<DL	0.04	20.8	16.1
		100	<DL	<DL	0.03	20.9	16.1
		105	<DL	<DL	0.03	20.9	16.2
		110	<DL	4.6	0.03	20.9	16.2

	Euxinic	130	<DL	15	<DL	21.1	16.4
		170	<DL	32	<DL	21.4	16.6
		250	<DL	85	<DL	21.7	16.8
		500	<DL	206	<DL	22.1	17.0
		1000	<DL	354	<DL	22.3	17.2
		2000	<DL	397	<DL	22.3	17.2

**2017**	Oxic	50	170	<DL	2.8	19.3	15.0
		55	115	<DL	3.6	19.5	15.1
		60	43	<DL	4.7	19.7	15.3
		70	5.9	<DL	3.0	20.2	15.6

	Suboxic	90	<DL	<DL	0.06	20.7	16.0

	Euxinic	130	<DL	<DL	<DL	21.2	16.4
		500	<DL	7.2	<DL	22.1	17.0
		1000	<DL	114	<DL	22.3	17.2
		1500	<DL	165	<DL	22.3	17.2
		2000	<DL	208	<DL	22.3	17.2

*mbsl, meter below sea level. HS^–^ measurements taken in 2017 have been deemed unreliable as they are lower than those in 2013, possibly due to Niskin bottle leakage. Hence the redox zones for the 2017 depths were determined based on the similarity in the oxygen, nitrate, salinity, and density values between 2013 and 2017. DL, detection limit. For list of detection limits see Sollai et al. (2018).*

Suspended particulate matter was collected on pre-ashed filters mounted on McLane WTS-LV *in situ* pumps (McLane Laboratories Inc., Falmouth, United Kingdom). In 2013 142-mm-diameter 0.7-μm pore size glass fiber GF/F filters (Pall Corporation) were used and in 2017 they were 0.3 μm GF75 filters (Advantec). Upon the recovery of the *in situ* pumps on the deck of the ship, the filters were immediately stored at −80°C until extraction. Physical parameters of the water column were recorded with a Sea-Bird SBE911C conductivity–temperature–depth (CTD) system equipped with a 24 × 12 L Niskin bottle rosette sampler. For the methods used for the measurement of dissolved oxygen (O_2_) and inorganic nutrients see Sollai et al. (2018).

### SPM Extraction and LC-MS Analysis

Lyophilized filters were extracted using a modified Bligh-Dyer procedure (similar to that described in Sturt et al., 2004) in batches of three alongside an extraction blank, consisting of pre-ashed glass fiber filter. Briefly, the cut-up filters were twice extracted ultrasonically for 10 min in a mixture of methanol, dichloromethane, and phosphate buffer (2:1:0.8, v:v) and the combined supernatants were phase-separated by adding additional dichloromethane and buffer to a final solvent ratio of 1:1:0.9 (v:v). The organic phase containing the IPLs was collected and the aqueous phase re-extracted three times with dichloromethane. All steps of the extraction were then repeated on the residue but with a mixture of methanol, dichloromethane, and aqueous trichloroacetic acid solution (TCA) pH 3 (2:1:0.8, v:v). Finally, the organic extracts were combined and dried under a stream of N_2_ gas. Before analysis the extract was redissolved in a mixture of MeOH:DCM (9:1, v:v) which contained two internal standards (IS), a platelet activating factor (PAF) standard (1-O-hexadecyl-2-acetyl-snglycero-3-phosphocholine) and a deuterated betaine lipid {1,2-dipalmitoyl-sn-glycero-3-O-4′-[N,N,N-trimethyl(d9)]-homoserine; Avanti Lipids}. Aliquots were filtered through 0.45 μm regenerated cellulose syringe filters (4 mm diameter; Grace Alltech). Extraction blanks were performed alongside the SPM extractions, using the same glassware, solvents and extraction methodology, but with no glass fiber or SPM material.

Analysis of SPM extracts was carried out using UHPLC-HRMS according to the reversed phase method of Wörmer et al. (2013) with the following modifications. We used an Agilent 1290 Infinity I UHPLC, equipped with thermostatted auto-injector and column oven, coupled to a Q Exactive Orbitrap MS with Ion Max source with heated electrospray ionization (HESI) probe (Thermo Fisher Scientific). Separation was achieved on an Acquity BEH C18 column (Waters, 2.1 × 150 mm, 1.7 μm) maintained at 30°C. The eluent composition was (A) MeOH/H_2_O/formic acid/14.8 M NH_3aq_ [85:15:0.12:0.04 (v:v)] and (B) IPA/MeOH/formic acid/14.8 M NH_3aq_ [50:50:0.12:0.04 (v:v)]. The elution program was: 95% A for 3 min, followed by a linear gradient to 40% A at 12 min and then to 0% A at 50 min, this was maintained until 80 min. The flow rate was 0.2 mL min^–1^. Positive ion HESI settings were: capillary temperature, 300°C; sheath gas (N_2_) pressure, 40 arbitrary units (AU); auxiliary gas (N_2_) pressure, 10 AU; spray voltage, 4.5 kV; probe heater temperature, 50°C; S-lens 70 V. Lipids were analyzed with a mass range of *m/z* 350–2000 (resolving power 70,000 ppm at *m/z* 200), followed by data-dependent tandem MS/MS (resolving power 17,500 ppm), in which the 10 most abundant masses in the mass spectrum were fragmented successively (stepped normalized collision energy 15, 22.5, 30; isolation width, 1.0 *m/z*). The Q Exactive was calibrated within a mass accuracy range of 1 ppm using the Thermo Scientific Pierce LTQ Velos ESI Positive Ion Calibration Solution. During analysis dynamic exclusion was used to temporarily exclude masses (for 6 s) in order to allow selection of less abundant ions for MS/MS.

It should be noted that IPL species have diverse degrees of ionization efficiencies (Van Mooy and Fredricks, 2010) and hence the peak areas, in response units, of different components do not necessarily reflect their actual relative abundance. However, this method allows for comparison between samples when analyzed together.

### Data Processing

Each SPM extract was analyzed in duplicate by UHPLC-HRMS, in sequence with extraction blanks (Bligh-Dyer extraction without SPM material) and analytical blanks (solvent injections on system between extract analyses). Extractions blanks were included in the subsequent data processing scheme, while analytical blanks were used for routine quality control checks but were not included in further data processing. For the Black Sea 2013 series, there were 30 analyses of SPM extracts (15 samples in duplicate) and 8 analyses of extraction blanks (4 extraction blanks in duplicate), totaling 38 analyses. In order to provide relevant information about background compounds and contamination, all extraction blanks were carried out using approximately the same solvent volumes as for the samples and extracts and were injected in the same injection volume. For the Black Sea 2017 series, there were 22 analyses of SPM extracts (11 samples in duplicate) and 8 analyses of extraction blanks (4 blanks in duplicate), totaling 30 analyses. For each UHPLC-HRMS analysis, the raw data files was converted to an.mz XML file using MSconvert of the ProteoWizard package (Chambers et al., 2012). The mzXML files were then processed using MZmine software (Version 2.34) (Pluskal et al., 2010). A signal threshold of 1 × 10^5^ was applied for MS^1^ mass detection within the mzXML files. The detected masses were used to build extracted ion chromatograms (EIC) with a minimum peak height of 1 × 10^6^ (AU) and a 3 ppm relative mass tolerance. Chromatographic deconvolution (separation of detected masses into individual peaks) was carried out with the “baseline cutoff” algorithm where the minimum peak height was 1 × 10^5^ AU and the maximum peak width was 5 min. Following the deconvolution, isotope peak grouping was carried out with a 3 ppm relative mass tolerance and a 0.2 min retention time tolerance followed by alignment of EICs with a 20 ppm mass tolerance and a 0.5 min retention time tolerance. Only aligned EICs containing a minimum of two isotope peaks and occurring in at least two samples were included. The next stage of processing removed duplicate peaks (within a window of 5 ppm mass and 0.4 min retention time). For each individual component, an MS^1^ peak area was recorded for each sample, which was then corrected for the amount of sea water filtered (in L).

**FIGURE 1:**
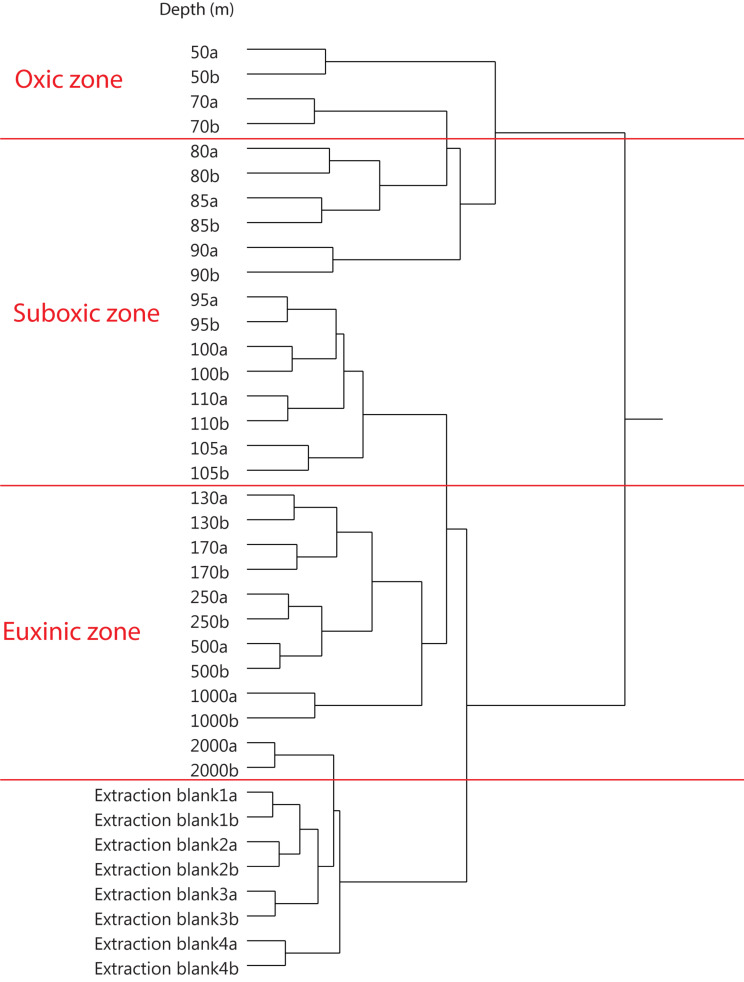
A preliminary analysis, using average-neighbor hierarchical clustering (Ward method) of the 38 UHPLC-HRMS analyses (Black Sea water column SPM from 2013) and associated extraction blanks. Clustered by similarity in distribution of lipidome components (*n* = 14,648), as extracted by MZmine software. Red annotations show the water column redox zones, as given in Table 1.

A preliminary statistical analysis of datasets was then carried out using Ward method average-neighbor hierarchical clustering (Ward, 1963) using JMP^®^ software (Version 14.2.0., SAS Institute Inc.). For the 2013 dataset, for example, the 38 analyses were clustered according to the similarity in their component distributions (Figure 1). All duplicate analyses clustered together, demonstrating the reproducibility of the UHPLC-HRMS analytical method and the MZmine component extraction. The SPM extracts also clustered in a relatively good accordance to the different redox zones where they were collected (Table 1) as indicated with red annotation in Figure 1. The duplicate SPM extracts from 2000 mbsl depth clustered most closely with the extraction blanks, indicative of the much lower concentration of organic matter at this depth.

For both datasets a blank subtraction method was subsequently carried out. For each component an average of the extraction blank peak areas was calculated (2013, *n* = 8; 2017, *n* = 8). This was then deducted from the average of the SPM extract peak areas (2013, *n* = 30; 2017, *n* = 20). While this led to the complete removal of many contaminants, some remained in the final dataset. When examining the most abundant components in the various clusters, eight known contaminants remained, mostly in the 2000 mbsl SPM extracts (Supplementary Table 1). It is possible these contaminants were introduced during sampling rather than extraction as they occurred in both duplicate samples. These eight contaminants were excluded from further analysis. In the 2013 dataset blank subtraction led to the complete removal of 996 components and in the 2017 dataset it was 562.

For the components remaining after blank extraction, an average value was calculated for each pair of duplicate SPM analyses. The resulting two datasets were then analyzed by two-way average-neighbor hierarchical clustering (using JMP^®^), again following the Ward method, according first to component distribution in samples and then two way clustered to component abundance across samples. This clustering was visualized in a two-dimensional dendrogram with peak area abundance shown with a color scale.

## Results and Discussion

### Untargeted Analysis of Black Sea SPM Collected in 2013

The Black Sea is the world’s largest permanently stratified anoxic basin. The SPM collected covered the full range of redox zones throughout the water column (Table 1): the oxic zone, the suboxic zone, which is defined as the zone where both oxygen and sulfide are below detection levels, and the euxinic zone where the water is both anoxic and sulfidic (Murray et al., 1999).

**FIGURE 2:**
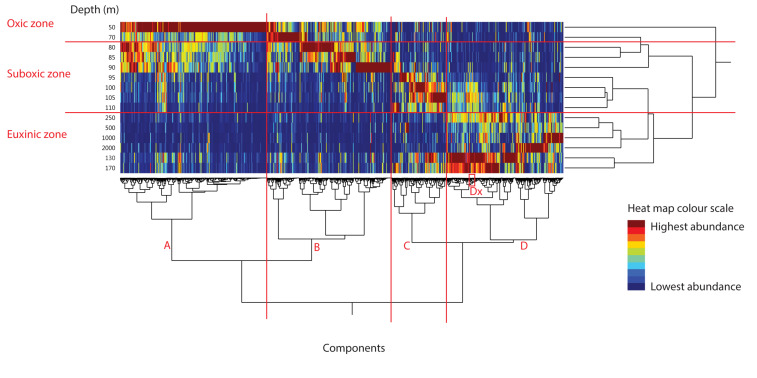
Heat map of the peak areas of 14,648 component in Black Sea SPM from June 2013 as extracted by MZmine software. Color scheme represents component’s depth profile, red, highest abundance; and blue, lowest abundance. SPM extracts clustered by similarity in component distribution on the vertical axis and components clustered by similarity of depth profile through water column on the horizontal axis. Red annotations show the water column redox zones, as given in Table 1.

Lipid extracts of SPM (*n* = 15) produced a final dataset of 14,648 components. This entire final dataset then underwent two-way average-neighbor hierarchical clustering, dendrogram construction and a heat map plotting using JMP^®^ software (Figure 2). The clustering of the SPM extracts (vertical axis, denoted by the depth at which the SPM was collected) is based on the distribution of all MS^1^ spectral components within the SPM extracts. The clustering of the components (horizontal axis) is based on the similarity of the abundance depth profiles for each component. Each individual depth profile in Figure 2 has a color scale (heat map, red, highest abundance of component in depth profile; blue, lowest abundance of component in depth profile). This visualization of both the similarity between SPM extracts and the similarity between the component depth profiles is a useful resource for a rapid examination of the dataset. Firstly, it reveals component clusters that are associated with specific niches, environmental variables, or organisms of interest. Secondly, it is clusters that contain a known component can be used to find other components with a similar distribution through the water column. Hereafter we give some examples of such examinations.

#### Component Clusters and Their Association With Specific Redox Zones

The vertical clustering generally followed the three redox zones (cf. Table 1) as marked (with red horizontal lines and red labels). The division between the redox zones was also visible in the distinct areas of component abundance maxima (red color) on the heat map, indicating a general division in the component signature of the SPM based on different chemical, and by extension, biological strata. The dendrogram of the 14,648 components (along the horizontal axis, Figure 2) showed four main clusters. We denoted these four large clusters as A–D (with red vertical lines and red labels). The four component clusters also correlated well with the different redox zones. Component cluster A (4846 components) corresponded most closely with the oxic zone (50 mbsl). Cluster B (4131 components) was most associated with the upper half of the suboxic zone and the lowest depth from the oxic zone (70–90 mbsl), while cluster C (1822 components) corresponded with the deeper part of the suboxic zone (95–110 mbsl). Component cluster D (3849 components) was most associated with the euxinic zone (130–2000 mbsl).

This division of the components into redox-defined niches of the Black Sea is in line with the earlier findings from traditional lipid analyses (Repeta and Simpson, 1991; Kuypers et al., 2003; Wakeham et al., 2003, 2007; Coolen et al., 2007; Schubotz et al., 2009; Becker et al., 2018b). The benefit of this non-targeted visualization approach, however, is that it is quick, unbiased, uses all mass spectral information and does not require the laborious integration of all compounds, nor the plotting of their individual depth profiles. Furthermore, the components, while dominated by lipids due to the extraction and analytical methods applied, are expected to contain many other biomolecules, expanding the search for potential biomarkers associated with microorganisms or processes of interest.

#### Abundant Components in Major Clusters

In order to examine the output of our component extraction and visualization method, we examined the 10 most abundant components in each of the four component clusters A–D (Table 2) in order to identify them and to compare the results with previous studies of the Black Sea lipidome (e.g., Neretin et al., 2007; Wakeham et al., 2007; Schubotz et al., 2009; Sollai et al., 2018). Identification followed traditional MS interpretation methods, looking at the accurate mass of the component and its MS/MS spectrum in comparison with published data. In the component cluster A (associated with the oxic zone), seven of the 10 most abundant components were triacylglycerols (TAG; Hsu and Turk, 1999). TAG lipids can form an important fraction of the total fatty acid inventory, and are utilized as storage lipids in algae and zooplankton (i.e., Guschina and Harwood, 2009; Becker et al., 2018a). Also abundant components in cluster A were two betaine lipids: diacylglycerylcarboxyhydroxymethylcholine (DGCC) and diacylglyceryltrimethylhomoserine (DGTS; Li et al., 2017). Betaine lipids have been found in a variety of eukaryotes and photosynthetic bacteria (Dembitsky, 1996) and DGTS in particular is found in green algae (Li et al., 2017), while DGCCs have been shown to be a common membrane lipid in Haptophyceae algae (Kato et al., 1996). Also present was the photosynthetic pigment chlorophyll-*a* (chl-*a*) (Bale et al., 2011; Table 2). The dominance of TAGs, betaines lipids and chl-*a* in the component cluster associated with the oxic (photic) zone are all in line with a dominance of phytoplankton. In fact, the western Black Sea experiences extensive phytoplankton blooms, especially during the summer period (Moncheva et al., 2001) when our sampling occurred. In a previous study of IPLs throughout the Black Sea water column, betaine lipids were found to be very abundant in the oxic zone but TAGs and chlorophylls were not reported owing to the applied chromatographic method (Schubotz et al., 2009).

**TABLE 2:** The most abundant components of cluster A–D in the Black Sea SPM extracts (2013 expedition).

**Component cluster**	**MS^1^ ion (*m/z*)**	**RT (min)**	**AEC**	**Δ mmu**	**Assignment**	**Diagnostic element in MS/MS**
**A** Oxic zone	822.7526	42.94	C_51_H_100_NO_6_^+^	1.9	TAG 14:0, 16:0, 18:1	523.4711, 549.4868, 577.5181
	850.7841	45.65	C_53_H_104_NO_6_^+^	1.7	TAG 16:0, 16:0, 18:1	551.5026, 577.5182
	848.7677	42.99	C_53_H_102_NO_6_^+^	2.5	TAG 14:0, 18:1, 18:1	549.4867, 603.5336
	794.7231	40.00	C_49_H_96_NO_6_^+^	0.1	TAG 14:0, 16:0, 16:1	521.4554, 523.4708, 549.4866
	876.7994	45.63	C_55_H_106_NO_6_^+^	2.0	TAG 16:0, 18:1, 18:1	577.5181, 603.5334
	800.6020	18.10	C_48_H_82_NO_8_^+^	1.5	DGCC-DAG 38:6	104.172, 132.1018
	922.7846	42.05	C_59_H_104_NO_6_^+^	1.2	TAG 16:0, 18:1, 22:6	577.5177, 623.5023, 649.5173
	893.5402	21.63	C_55_H_73_MgN_4_O_5_^+^	2.4	Chlorophyll-*a*	555.2230, 583.2182, 615.2420
	894.7527	39.20	C_57_H_100_NO_6_^+^	1.8	TAG 16:0, 16:1, 22:6	549.4865, 621.4888, 623.5028
	732.5770	17.09	C_44_H_78_NO_7_^+^	1.0	DGTS-DAG 34:4	100.1121, 144.1018

**B** Upper suboxic zone	848.7679	43.41	C_53_H_102_NO_6_^+^	2.3	TAG 16:0, 16:0, 18:2	551.5021, 575.5021
	874.7839	43.47	C_55_H_104_NO_6_^+^	1.9	TAG 16:0, 18:1, 18:2	575.5021, 577.51788, 601.5178
	872.7682	41.34	C_55_H_102_NO_6_^+^	1.9	TAG 16:0, 18:2, 18:2	575.5021, 599.5021
	820.7369	40.65	C_51_H_98_NO_6_^+^	2.0	TAG 14:0, 16:0, 18:2	523.4711, 547.4712, 575.522
	820.7369	40.17	C_51_H_98_NO_6_^+^	1.9	TAG 14:0, 16:1, 18:1	521.4556, 549.4868, 575.5023
	868.7396	39.07	C_55_H_98_NO_6_^+^	0.7	TAG 14:0, 16:0, 22:6	523.4713, 595.4721, 623.5020
	896.7698	41.98	C_57_H_102_NO_6_^+^	0.4	TAG 16:0, 16:0, 22:6	551.5026, 623.5023
	806.5675	18.49	C_46_H_81_NO_8_P^+^	1.9	PC-DAG (38:6)	184.0731
	866.7223	35.45	C_55_H_96_NO_6_^+^	0.9	TAG 16:0, 18:3, 18:4	573.4866, 593.4556
	682.5621	17.17	C_40_H_76_O_7_N^+^	0.5	DGTS-DAG 30:1	100.1112, 144.1016

**C** Lower suboxic zone	688.4911	16.97	C_37_H_71_NO_8_P^+^	0.1	PE-DAG (16:1,16:1)	Loss of 141.0186
	818.7211	37.25	C_51_H_96_NO_6_^+^	2.2	TAG 16:1, 16:1, 16:1	547.4714
	1302.3194	62.83	C_86_H_173_O_6_^+^	3.3	GDGT-0	1302.3207
	1292.2445	66.46	C_86_H_163_O_6_^+^	0.1	Crenarchaeol	1292.2433
	470.2533	5.38	C_27_H_36_NO_6_^+^	0.4	Unassigned component I	113.0598, 247.1324
	528.3747	28.79	C_40_H_48_^+^	0.4	Isorenieratene	133.1010, 436.3115
	547.4717	17.00	C_35_H_63_O_4_^+^	0.4	AEG O-16:2, 16:2	237.2202
	549.4870	18.73	C_35_H_65_O_4_^+^	0.7	AEG O-16:2, 16:1	237.2197
	702.5065	16.99	C_38_H_73_O_8_NP^+^	0.3	MMPE-DAG 16:1, 16:1	Loss of 155.0347
	738.5281	16.48	C_38_H_77_NO_10_P^+^	0.2	PG-DAG 16:1, 16:0	Loss of 189.0395

**D** Euxinic zone	352.3207	6.82	C_22_H_42_NO_2_^+^	0.3	Fatty acid 22:3	317.2832, 335.2938
	690.5069	18.70	C_37_H_73_NO_8_P^+^	0.0	PE-DAG 16:1, 16:0	Loss of 141.0206
	693.5430	18.75	C_38_H_78_O_8_P^+^	0.1	PG-DEG o-16:0, O-16:1	Loss of 172.0090
	676.5273	19.88	C_37_H_75_NO_7_P^+^	0.3	PE-AEG 32:1	Loss of 141.0196
	660.5344	19.41	C_37_H_75_NO_6_P^+^	1.7	PE-DEG O-16:1, O-16:1	Loss of 43.0423
	928.8001	23.90	C_55_H_110_NO_7_S^+^	0.4	Acetylsulfono-1-deoxyceramide	310.3463, 868.777
	900.7672	21.73	C_53_H_106_NO_7_S^+^	1.2	Acetylsulfono-1-deoxyceramide	282.3150, 840.7456
	554.5495	23.71	C_35_H_72_NO_3_^+^	1.2	DEG O-16:0, O-16:2	279.2668, 315.2887
	650.5476	20.44	C_36_H_77_NO_6_P^+^	0.7	MMPE-DEG 30:0	58.0659
	662.5489	21.51	C_37_H_77_NO_6_P^+^	0.6	PE-DEG O-16:0,O-16:1	Loss of 43.0417

*Known LCMS contaminants were excluded (see text for details). AEC, assigned elemental composition. Δ = (measured mass − calculated mass) × 1000. mmu, milli mass unit. For abbreviations of component assignment see text. Numbers in component assignment indicate total alkyl carbons (not including polar head group or glycerol moiety) and number of double bond equivalents (cf. Liebisch et al., 2013).*

In cluster B (associated with the upper part of the suboxic zone, 70–90 mbsl), the eight of the 10 most abundant components were also TAG lipids. A DGTS betaine lipids was also in the most abundant components of this cluster along with a phosphatidylcholine (PC) lipid with a highly unsaturated diacyl glycerol (DAG) core: PC-DAG 38:6. PCs containing long chain highly unsaturated acyl moieties are associated with phytoplankton (Brett and Müller−Navarra, 1997) and have been observed previously in the oxic zone of the Black Sea (Schubotz et al., 2009). These results indicate that the upper part of the suboxic zone was still dominated by phytoplankton-derived lipids.

The 10 most abundant components in cluster C (associated with the lower half of the suboxic zone; Table 2) included three DAG-phospholipids: a phosphoethanolamine (PE) and a monomethyl PE (MMPE) both with two 16:1 fatty acids, and a phosphoglycerol (PG)-DAG with 16:0,16:1 fatty acids. PE-IPLs are frequently the main lipid component of bacterial membranes. Schubotz et al. (2009) also found diacyl phospholipids to be characteristic of the suboxic zone of the Black Sea. The 2nd most abundant component was a TAG (with 16:1, 16:1, 16:1 fatty acids). As well as being storage lipids in living algae and zooplankton, TAGs are present in sinking particles (Wakeham, 1985) and hence their abundance here, well below the oxic zone, may be due to sinking material, including algal cells and zooplankton fecal pellets (Hay et al., 1990; Bologa et al., 1999; Moncheva et al., 2001; Wakeham et al., 2007). Two other abundant components in cluster C were acyl/ether glycerol lipids (termed AEGs; Sturt et al., 2004) without a polar head group: both with 16:2 ether bound chains and with a 16:1 fatty acid and a 16:2 fatty acid, respectively. AEGs have been reported mainly in anaerobic bacteria, although with some exceptions in aerobic bacteria, and are thought to improve cell resistance, relative to DAGs, to extreme external conditions (Grossi et al., 2015).

Also among the top six components in cluster C were two core glycerol dialkyl glycerol tetraethers (GDGTs): GDGT-0 and crenarchaeol. These archaeal membrane lipids are *sn*-2,3-dialkyl diglycerol tetraethers with two glycerol moieties connected by two C_40_ isoprenoid chains which contain 0–8 cyclopentane moieties (i.e., GDGT-n, where *n* is the number of cyclopentane moieties) (Schouten et al., 2013). To date, crenarchaeol (which has four cyclopentane moieties and one cyclohexane moiety) has only been associated with the archaeal phylum Thaumarchaeota and hence is considered to represent a specific biomarker for members of this phylum (e.g., Sinninghe Damsté et al., 2002b; Schouten et al., 2013; Bale et al., 2019a). GDGT-0 is a more cosmopolitan GDGT, produced by representatives of the Thaumarchaeota, Crenarchaeota, and Euryarchaeota (Villanueva et al., 2014). Previous studies of archaeal lipids in the Black Sea water column also reported both GDGT-0 and crenarchaeol throughout the water column (Wakeham et al., 2003; Coolen et al., 2007; Sollai et al., 2018). Sollai et al. (2018) found a maximum in archaeal 16S rRNA gene copies at 105 m, in the lower part of the suboxic zone, mainly attributed to *Nitrosopumilus* species, many of which are known to produce GDGT-0 and crenarchaeol as their dominant GDGTs (Sinninghe Damsté et al., 2002a; Schouten et al., 2008; Pitcher et al., 2011; Qin et al., 2015; Elling et al., 2017). Another abundant component at this depth was isorenieratene, a specific carotenoid of the photosynthetic sulfur bacteria *Chlorobiaceae* (Repeta, 1993; Koopmans et al., 1996; Sinninghe Damsté et al., 2001; Brocks et al., 2005). That isorenieratene is found in component cluster C, associated with the lower suboxic zone, is to be expected, as *Chlorobiaceae* perform photosynthesis using sulfide, and hence require photic zone anoxia, as has been shown to occur in the suboxic zone of the Black Sea (Repeta et al., 1989; Repeta and Simpson, 1991; Overmann et al., 1992; Becker et al., 2018b).

Finally, one of the most abundant components in cluster C was an unassigned component (I), with *m/z* 470.2520 (Table 2; cf. Supplementary Figure 1A for MS/MS spectrum). The presence of this unassigned component in the 10 most abundant components of cluster C highlights that our untargeted method has the potential to identify novel compounds associated with specific redox zones and by extension, specific groups of organisms or their activity. Using traditional MS data interpretation approaches, this component would have very likely been overlooked as it occurred at only a few depths. Such components would be interesting targets for future rigorous identification approaches or for matching with the continuously increasing number of online spectral libraries.

In component cluster D, associated with the euxinic zone, the most abundant feature was a 22:3 fatty acid without a headgroup or glycerol moiety, followed by a PE-DAG with 16:1, 16:0 fatty acids, and a PE-AEG 32:1. A previous study described PE-AEG as an important component of the lipidome at the oxic zone/oxygen minimum zone (OMZ) transition in the eastern tropical North Pacific (Schubotz et al., 2018). Also in the 10 most abundant components were multiple diether glycerol (DEG)-based lipids: PE-DEG with O-16:1 and O-16:0 (denoting ether-bound alkyl chain), MMPE-DEG 30:0, a PG-DEG O-16:0, O-16:1 and a DEG O-16:0, O-16:2 without a polar headgroup. Diether lipids have been detected in a limited number of bacteria (e.g., Grossi et al., 2015) but their (core lipid) abundance in the Black Sea water column has been shown to correlate with sulfate reducing bacteria (SRB; Neretin et al., 2007). Two components, tentatively assigned as sulfate-1-deoxyceramides (Ding et al., 2021), were also among most abundant components in cluster D. Ding et al. (2021) hypothesize that, as bacterial sphingolipids are mainly produced by *Bacteroidetes* and selected *Proteobacteria* (Heaver et al., 2018) and because certain *Bacteroidetes* from the Black Sea are known to produce capnines (Bale et al., 2020; Yadav et al., 2020), sulfono-analogs of sphinganine (Godchaux and Leadbetter, 1980), that these novel sulfate-1-deoxyceramides, may also be produced by anaerobic heterotrophs related to *Bacteroidetes*.

In summary, our non-targeted data analysis resulted in clusters of components associated with specific depths. The zonation of these components (the major ones being lipids) due to microbial niches is in agreement with previous studies (Wakeham et al., 2007; Schubotz et al., 2009), however, our approach is rapid and unbiased and includes all components within the analytical window whether they have been identified or not.

#### Rapid Clustering of Lipids Based on Depth Profile Similarity

The LCMS data visualization method we have presented here is useful to examine clusters that contain a known component, in order to find other components with the same distribution through the water column. Here, we demonstrate this by examining other components in a subcluster containing the sulfate-1-deoxyceramide with *m/z* 928.8001 (cluster D, associated with the euxinic zone, Table 2). As discussed in Ding et al. (2021), this novel 1-deoxysphingolipids may be produced by an as-yet uncultured anaerobic heterotroph related to *Bacteroidetes*. We examined components with a very similar depth profile, that may be associated with the same microbial producer or with producers that live in the same environmental niche. At a higher level of clustering, the sulfate-1-deoxyceramide with *m/z* 928.8001 was in a cluster of 24 components (Dx; as indicated in Figure 2). We present here the 10 most abundant components in this subcluster (Dx) (Table 3). All 10 components followed a similar abundance depth profile with a distinct maximum at 130 m. Other than the sulfate-1-deoxyceramide with *m/z* 928.8001 (Ding et al., 2021), no other components in Dx were in the list of top 10 most abundant components in cluster D.

**TABLE 3:** The most abundant components of cluster Dx in the Black Sea SPM extracts (2013 expedition).

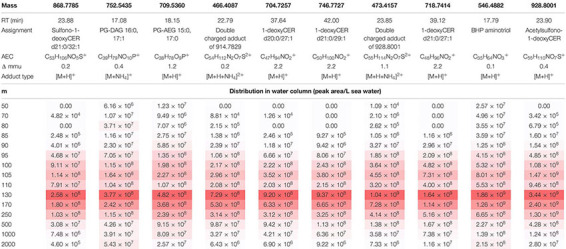

*1-deoxyCER, 1-deoxyceramide; AEC, assigned elemental composition. Δ = (measured mass − calculated mass) × 1000. mmu, milli mass unit. For abbreviations of component assignment see text. Numbers in component assignment indicate total alkyl carbons (not including polar head group or glycerol moiety) and number of double bond equivalents (cf. Liebisch et al., 2013). Color scheme represents component’s depth profile, red, highest abundance and no color, lowest abundance.*

The second most abundant component was assigned as aminotriol bacteriohopanepolyol (BHP) based on its accurate mass and fragmentation spectra (Hopmans et al., 2021). This BHP is relatively ubiquitous in marine systems (Talbot et al., 2014; Rush et al., 2016) but has been specifically associated with aerobic methanotrophic bacteria (Cvejic et al., 2000) and with SRB (Blumenberg et al., 2012). At its maxima at 130 mbsl, at the top of the euxinic zone, it is most probably associated with SRB. Three components were 1-deoxyceramides and two were sulfono-1-deoxyceramides (as per their assignment in Ding et al., 2021). Also present were two PGs, one with a DAG 33:1 core and one with a 32:0 core. Two of the components listed, at *m/z* 473.4157 and 466.4087 were artifacts of analysis, doubly charged acetylsulfono-1-deoxyceramides (with [M+H]^+^ at *m/z* 928.8001 and 914.7830, respectively). This was clear due to the 0.5 Da spacing in their isotope pattern (Supplementary Figure 2).

Here, we show that the selection of a subcluster of components around a component of interest is straightforward and quick and allows for rapid associations between different components to be made, based on their specific depth profiles, rather than their chemical composition.

### Comparison Between SPM Collected in 2013 and 2017

**FIGURE 3:**
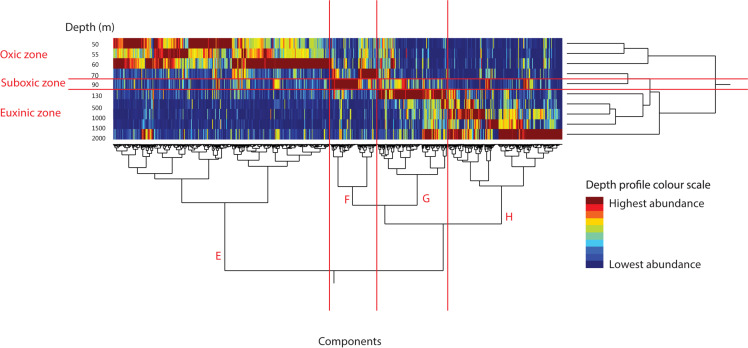
Heat map of the peak area of 12,372 components in Black Sea SPM from March 2017 as extracted by MZmine software. Color scheme represents component’s depth profile, red, highest abundance; and blue, lowest abundance. SPM extracts clustered by similarity in component distribution on the vertical axis and components clustered by similarity of depth profile through water column on the horizontal axis. Red annotations show the water column redox zones, as given in Table 1.

In order to examine the robustness of our method for distinguishing the lipidome associated with different depths of the Black Sea water column, we followed the same method of component extraction and visualization on SPM extracts from the Black Sea water column, collected in March 2017 at a station located ∼110 km from that of 2013 cruise. SPM was collected at an earlier time in the year (March versus June), with a different distribution of sampling depths (more sampling in oxic zone, less in suboxic zone, Table 1), and using a smaller (nominal) pore size of filter (0.3 versus 0.7 μm). We followed the same data extraction and data processing procedure as described above for the 2013 sample set. The heat map generated from these data again shows division in the lipidome (Figure 3), supported by the vertical clustering, generally in line with the three redox zones. The dendrogram of the 12,372 components was again made up of four distinct clusters E–H (Figure 3), which corresponded with the redox zones. Component cluster E (6006 components) corresponded with the oxic zone. Cluster F (1292 components) with the suboxic zone, and again included the 70 mbsl depth SPM, from the base of the oxic zone. Cluster G (1941 components) corresponded with the uppermost sampling depth from the euxinic zone (130 mbsl), while cluster G (3133) with the remainder of the depths in the euxinic zone.

We again examined the 10 most abundant components (Table 4) in component clusters E–H (Figure 3). In cluster E, associated with the oxic zone, six of the 10 most abundant components were TAG lipids, similar to the 2013 dataset (Table 2). Two more abundant components were the chl-*a* alteration product pheophytin-*a* and the epimer of pheophytin-*a* (C-13^2^ diastereomer). Demetalation of chl-*a* to produce pheophytin-*a* occurs readily when chl-*a* is free from its stable protein complex. While pheophytin can be formed naturally, e.g., during cell senescence or grazing (Louda et al., 1998, 2002; Bale et al., 2011), it is likely that in this study it represents an artifact of sampling and extraction (transformation of chl-*a*) as the sample collection and extraction were not optimized for pigment analysis. The presence of pheophytin-*a*, whether a natural occurring compound or a proxy for chlorophyll is not surprising as the oxic photic zone of the western Black Sea experiences extensive phytoplankton blooms (Moncheva et al., 2001). Pheophytin-*a* was not among the most abundant 10 components in cluster A (although chl-*a* was the 8th most abundant component). The same betaine lipid, DGTS 34:4 as was seen in cluster A from 2013 was among the 10 most abundant components in cluster E from 2017. Finally, an unassigned component (II), with *m/z* 547.2703 (Table 2; cf. Supplementary Figure 1B for MS/MS spectrum) was among the most abundant components in this cluster.

**TABLE 4:** The most abundant components of cluster E–H in the Black Sea SPM extracts (2017 expedition).

**Feature cluster**	**MS^1^ ion (*m/z*)**	**RT (min)**	**AEC**	**Δ mmu**	**Assignment**	**Diagnostic element in MS/MS**
**E** Oxic zone	871.5724	30.09	C_55_H_75_N_4_O_5_^+^	0.8	Pheophytin-a	533.2540, 593.2752
	872.7695	41.43	C_55_H_102_NO_6_^+^	0.7	TAG 16:0, 18:1, 18:3	573.4781, 575.5184, 599.5032
	850.7850	45.54	C_53_H_104_NO_6_^+^	0.8	TAG 16:0, 16:0, 18:1	551.5029, 577.5185
	822.7539	42.86	C_51_H_100_NO_6_^+^	1.9	TAG 14:0, 16:0, 18:1	523.4715, 549.4873, 577.5181
	871.5766	31.31	C_55_H_75_N_4_O_5_^+^	0.5	Pheophytin-a epimer	533.2540, 593.2752
	866.7228	36.04	C_55_H_96_NO_6_^+^	0.4	TAG 16:0, 18:3, 18:4	571.4714, 573.4869, 593.4556
	870.7528	39.31	C_55_H_100_NO_6_^+^	1.7	TAG 16:0, 18:2, 18:3	573.4781, 575.5026, 597.4870
	732.5772	17.19	C_44_H_78_NO_7_^+^	0.1	DGTS-DAG 34:4	100.1126, 144.1018
	547.2703	13.05			Unassigned II	
	846.7538	41.51	C_53_H_100_NO_6_^+^	0.7	TAG 16:0, 16:0, 18:4	551.5032, 573.4871

**F** Oxic/suboxic zone interface	688.4908	17.02	C_37_H_71_NO_8_P^+^	0.4	PE-DAG 16:1,16:1	Loss of 141.0200
	690.5069	18.77	C_37_H_73_NO_8_P^+^	0.0	PE-DAG 16:1,16:0	Loss of 141.0197
	792.7071	37.12	C_49_H_94_NO_6_^+^	0.3	TAG 14:0, 16:1 16:1	521.4560, 547.4716
	746.5770	19.45	C_41_H_80_NO_10_^+^	0.7	MG-DAG 16:0 16:1	Loss of 197.0786
	549.4879	21.52	C_35_H_65_O_4_^+^	0.2	AEG 32:3	219.2103, 237.2207, 239.23677
	818.7224	37.77	C_51_H_96_NO_6_^+^	0.8	TAG 16:1, 16:1 16:1	547.4718
	582.5097	19.52	C_35_H_68_NO_5_^+^	0.5	DAG 16:1 16:1	311.2578, 547.4722
	744.5592	17.51	C_41_H_78_NO_10_^+^	2.9	MG-DAG 16:1 16:1	Loss of 197.0895
	818.7205	37.19	C_51_H_96_NO_6_^+^	2.7	TAG 16:1, 16:1, 16:1	547.4719
	549.4868	19.18	C_35_H_65_O_4_^+^	0.9	AEG 32:3	219.2104, 237.2206, 239.2355

**G** Upper euxinic (130 mbsl)	693.5430	18.54	C_38_H_78_O_8_P^+^	0.1	PG-DEG O-16:0, O-16:1	Loss of 172.0110
	662.4760	16.72	C_35_H_69_NO_8_P^+^	0.4	PE-DAG 14:0, 16:1	Loss of 141.0189
	676.5273	19.90	C_37_H_75_NO_7_P^+^	0.3	PE-AEG 32:1	Loss of 141.0164
	549.4872	18.72	C_35_H_65_O_4_^+^	0.6	AEG 32:3	237.2214, 239.2381
	523.4715	15.65	C_33_H_63_O_4_^+^	0.2	AEG 30:2	225.2206
	712.6088	20.89	C_42_H_82_NO_7_^+^	0.2	DGTA-DAG 32:0	236.14897
	650.5120	18.87	C_35_H_73_NO_7_P^+^	0.1	PE-AEG 30:0	Loss of 141.0190
	563.5034	17.45	C_36_H_67_O_4_^+^	0.0	AEG 33:3	233.2260, 239.2367, 251.2368
	577.5192	18.19	C_37_H_69_O_4_^+^	0.2	AEG 35:3	239.2370, 265.2525
	512.1589	13.04			Unassigned III	

**H** Euxinic zone	746.5558	16.19	C_44_H_76_NO_8_^+^	0.7	DGCC-DAG 16:0, 18:5	104.1073, 132.1019
	650.5482	20.49	C_36_H_77_NO_6_P^+^	0.1	MMPE-DEG 30:0	58.0660
	774.6025	21.45	C_43_H_85_NO_8_P^+^	0.2	PC-DAG 35:1	184.0733
	636.5323	20.42	C_35_H_75_NO_6_P^+^	0.4	PE-DEG O15:0, O-15:0	Loss of 43.0414
	662.5482	21.42	C_37_H_77_NO_6_P^+^	0.1	PE-DEG O-16:0, O-16:1	Loss of 43.0415
	667.5283	17.69	C_36_H_76_O_8_P^+^	1.1	PG-DEG O-15:0, O-15:0	Loss of 172.0114
	730.2444	16.03			Unassigned IV	237.0791
	513.5240	25.06	C_33_H_69_O_3_^+^	0.1	DEG O-15:0, O-15:0	285.2785, 303.2890
	660.5344	19.42	C_37_H_75_NO_6_P^+^	1.7	PE-DEG O-16:1, O-16:1	Loss of 43.0418
	688.5651	22.57	C_39_H_79_NO_6_P^+^	1.1	PE-DEG O-17:1, O-17:1	Loss of 43.0396

*Known LCMS contaminants were excluded (see text for details). AEC, assigned elemental composition. Δ = (measured mass − calculated mass) × 1000. mmu, milli mass unit. For abbreviations of component assignment see text. Numbers in component assignment indicate total alkyl carbons (not including polar head group or glycerol moiety) and number of double bond equivalents (cf. Liebisch et al., 2013).*

Cluster F, associated with the suboxic zone (Table 4), included three TAG lipids, a similar result to that of 2013, where TAG lipids remained abundant below the oxic zone (Table 2). Also abundant were several DAG lipids: PE-DAG 16:0, 16:1 and 16:1, 16:1. In the 2013 dataset, similar PE-DAGs were associated with the lower suboxic zone (Table 2). Two monogalactosyl [MG]-DAGs (commonly referred to as a MGDG) with 16:0 and 16:1 fatty acids were dominant components in cluster F. MGDGs are predominately associated with eukaryotic chloroplasts and cyanobacteria, although they can also be produced by non-photosynthetic bacteria (Hölzl and Dörmann, 2007; Popendorf et al., 2011), including sulfate-reducing bacteria when phosphate concentrations are below 20 μM (Bosak et al., 2016). This component was not listed among the top 10 components in any cluster from 2013. A DAG comprising of two 16:1 fatty acids and no polar head group was also among the abundant components of this cluster as were two isomers of AEG 32:3 without a polar head group (Table 4). AEG 32:3 was similarly abundant in the 2013 cluster associated with the lower suboxic zone (Table 2).

In cluster G, which was associated most closely with the SPM from 130 mbsl (the upper euxinic zone), among of the most abundant component was a DGTS betaine lipid with a DAG 32:0 core, whereas in the 2013 a similar DGTS-DAG (30:1) was associated with the upper suboxic zone. The only other abundant DAG-based lipid in the top 10 components from this cluster was PE-DAG 14:0, 16:1. Similar PE-DAGs were present there in the 2013 clusters associated with the lower suboxic zone and the euxinic zone. PE-AEG 30:0 and 32:1 were also dominant in this cluster, of which the same or similar components had been associated with the euxinic zone from 2013 (which included 130 mbsl; Table 2). Also abundant were an AEG 32:3 (a later eluting isomer to the AEG 32:3 seen in cluster F), AEG 30:2, 33:3, and 35:3. Similar components were associated with the lower suboxic zone of the 2013 dataset. Only one DEG-based lipid was among the 10 most abundant in cluster G, PG-DEG with a 16:1 and 16:0 ether bound chain as had been seen in the euxinic zone-associated cluster from 2013. Finally, an unassigned component (III), *m/z* 512.1589 (Table 4; cf. Supplementary Figure 1C for MS/MS spectrum).

In cluster H, associated with the remainder of the euxinic zone, among the 10 most abundant component were two DAG-based lipids, a betaine lipid, DGCC-DAG with a 16:0 and 18:5 fatty acid and PC-DAG 35:1. Neither DGCCs nor PC-DAGs were seen in the euxinic zone cluster from 2013 and both are associated with photosynthetic organisms. Their presence at depth in 2017 may be due to sinking algal material (typically in fecal pellets or marine snow) present in the euxinic zone as particles slowly descend to the sea floor. The majority of the 10 most abundant components in this cluster were PE and MMPE DEG lipids with O-15:0, O-16:0, and O-16:1 ether bound chains, as was also observed in the euxinic zone-associated cluster from 2013. MMPE-DEG 30:0 has previously be postulated to be associated with sulfate-reducing bacteria in the anoxic zone of the Black Sea (Schubotz et al., 2009). Another abundant component of cluster H was a PG-DEG O-15:0, O-15:0. A similar component, PG-DEG O-16:0, O-16:1, occurred also in the 2013 euxinic zone cluster. Also, DEG O-15:0, O-15:0 without a polar head group was abundant. Another unassigned component (IV) was also found in this cluster, with *m/z* 730.2444 (Table 2; cf. Supplementary Figure 1D for MS/MS spectrum).

Overall, the UHPLC-HRMS component extraction and visualization method presented here provided, for both the 2013 and 2017 SPM datasets, a similar picture of a stratified lipidome, in accordance with the three main redox zones. There were many similarities between the two sample sets, for example the clusters associated with the oxic zone were in both years dominated by TAG lipids. The differences that were observed are probably attributable to the different distribution of sampling depths between the years (more sampling in of the oxic zone in 2017, more of the suboxic zone in 2013; Table 1), which led to a different clustering of depths. The similarity in the dominant components highlights the similarity in the Black Sea lipidome over time and hence the stability of the microbial niches in this system.

## Conclusion

We used an untargeted UHPLC-HRMS data analysis approach to visualize components in the SPM from the Black Sea water column collected in June 2013. This revealed distinct clusters of known and unknown components, dominated by lipids, associated with specific depths. The approach allows for a rapid and unbiased view of the lipidome of an environment and for the identification of unknown lipids which potentially contain important ecological information. E.g., on (uncultivated) microbes. A second dataset from March 2017 provided similar results in the suboxic and euxinic zones. This rapid untargeted data visualization approach unlocks a hidden potential in UHPLC-HRMS data and provides a framework for further lipidomic method development which includes the utilization of MS/MS spectra (Ding et al., 2021).

## Data Availability Statement

The raw data supporting the conclusions of this article will be made available by the authors, without undue reservation.

## Author Contributions

NB and SD carried out the data analyses. NB wrote the manuscript. CB carried out the extractions and lipid analysis. EH initiated the research theme and oversaw the lipid identification. LV led 2017 sample collection and provided the ecological overview. MGIA and AH provided the methodological support. SS and JSSD supervised the study. All authors contributed to the article and approved the submitted version.

## Conflict of Interest

The authors declare that the research was conducted in the absence of any commercial or financial relationships that could be construed as a potential conflict of interest.

## Publisher’s Note

All claims expressed in this article are solely those of the authors and do not necessarily represent those of their affiliated organizations, or those of the publisher, the editors and the reviewers. Any product that may be evaluated in this article, or claim that may be made by its manufacturer, is not guaranteed or endorsed by the publisher.

## Supplementary Material

Supplementary Figure 1 UHPLC-HRMS/MS spectra of unassigned components **(A)** I, **(B)** II, **(C)** III, and **(D)** IV. Mmu, milli mass unit. Δ mmu = (measured mass − calculated mass) × 1000.

Supplementary Figure 2 Partial MS^1^ spectra of double charged components listed in Table 3.

## References

[B1] Bale N. J. Airs R. L. Llewellyn C. A. (2011). Type I and Type II chlorophyll-a transformation products associated with algal senescence. *Org. Geochem.* 42 451–464. 10.1016/j.orggeochem.2011.03.016

[B2] Bale N. J. Hopmans E. C. Schoon P. L. de Kluijver A. Downing J. A. Middelburg J. J. (2016). Impact of trophic state on the distribution of intact polar lipids in surface waters of lakes. *Limnol. Oceanogr.* 61 1065–1077. 10.1002/lno.10274

[B3] Bale N. J. Koenen M. Yadav S. Hopmans E. C. Villanueva L. Sinninghe Damsté J. S. (2020). Diagnostic amide products of amino lipids detected in the microaerophilic bacteria Lutibacter during routine fatty acid analysis using gas chromatography. *Organic Geochem.* 144:104027. 10.1016/j.orggeochem.2020.104027

[B4] Bale N. J. Palatinszky M. Rijpstra W. I. C. Herbold C. W. Wagner M. Sinninghe Damsté J. S. (2019a). Membrane Lipid Composition of the Moderately Thermophilic Ammonia-Oxidizing Archaeon “Candidatus Nitrosotenuis uzonensis” at Different Growth Temperatures. *Appl. Environ. Microbiol.* 85 1332–1319. 10.1128/AEM.01332-19 31420340 PMC6805073

[B5] Bale N. J. Sorokin D. Y. Hopmans E. C. Koenen M. Rijpstra W. I. C. Villanueva L. (2019b). New insights into the polar lipid composition of extremely halo(alkali)philic euryarchaea from hypersaline lakes. *Front. Microbiol.* 10:377. 10.3389/fmicb.2019.00377 30930858 PMC6423904

[B6] Bauersachs T. Compaore J. Hopmans E. C. Stal L. J. Schouten S. Sinninghe Damsté J. S. (2009). Distribution of heterocyst glycolipids in cyanobacteria. *Phytochemistry* 70 2034–2039. 10.1016/j.phytochem.2009.08.014 19772975

[B7] Becker K. W. Collins J. R. Durham B. P. Groussman R. D. White A. E. Fredricks H. F. (2018a). Daily changes in phytoplankton lipidomes reveal mechanisms of energy storage in the open ocean. *Nat. Commun.* 9:5179. 10.1038/s41467-018-07346-z 30518752 PMC6281602

[B8] Becker K. W. Elling F. J. Schröder J. M. Lipp J. S. Goldhammer T. Zabel M. (2018b). Isoprenoid Quinones Resolve the Stratification of Redox Processes in a Biogeochemical Continuum from the Photic Zone to Deep Anoxic Sediments of the Black Sea. *Appl. Environ. Microbiol.* 84 2736–2717. 10.1128/AEM.02736-17 29523543 PMC5930376

[B9] Belt S. T. Massé G. Rowland S. J. Poulin M. Michel C. LeBlanc B. (2007). A novel chemical fossil of palaeo sea ice: IP25. *Organic Geochem.* 38 16–27. 10.1016/j.orggeochem.2006.09.013

[B10] Blumenberg M. Hoppert M. Krüger M. Dreier A. Thiel V. (2012). Novel findings on hopanoid occurrences among sulfate reducing bacteria: Is there a direct link to nitrogen fixation? *Organic Geochem.* 49 1–5. 10.1016/j.orggeochem.2012.05.003

[B11] Bologa A. S. Frangopol P. T. Vedernikov V. I. Stelmakh L. V. Yunev O. A. Yilmaz A. (1999). “Distribution of Planktonic Primary Production in the Black Sea,” in *Environmental Degradation of the Black Sea: Challenges and Remedies* NATO Science Series, eds Beşiktepe S. T. Ünlüata Ü Bologa A. S. (Dordrecht: Springer Netherlands), 131–145. 10.1007/978-94-011-4568-8_9

[B12] Bosak T. Schubotz F. Santiago-Torio A. de, Kuehl J. V. Carlson H. K. (2016). System-Wide Adaptations of Desulfovibrio alaskensis G20 to Phosphate-Limited Conditions. *PLoS One* 11:e0168719. 10.1371/journal.pone.0168719 28030630 PMC5193443

[B13] Brandsma J. Hopmans E. C. Brussaard C. P. D. Witte H. J. Schouten S. Sinninghe Damsté J. S. (2012). Spatial distribution of intact polar lipids in North Sea surface waters: Relationship with environmental conditions and microbial community composition. *Limnol. Oceanogr.* 57 959–973. 10.4319/lo.2012.57.4.0959

[B14] Brett M. Müller−Navarra D. (1997). The role of highly unsaturated fatty acids in aquatic foodweb processes. *Freshw. Biol.* 38 483–499. 10.1046/j.1365-2427.1997.00220.x

[B15] Brocks J. J. Love G. D. Summons R. E. Knoll A. H. Logan G. A. Bowden S. A. (2005). Biomarker evidence for green and purple sulphur bacteria in a stratified Palaeoproterozoic sea. *Nature* 437 866–870. 10.1038/nature04068 16208367

[B16] Chambers M. C. Maclean B. Burke R. Amodei D. Ruderman D. L. Neumann S. (2012). A cross-platform toolkit for mass spectrometry and proteomics. *Nat. Biotechnol.* 30 918–920. 10.1038/nbt.2377 23051804 PMC3471674

[B17] Collins J. R. Edwards B. R. Fredricks H. F. Van Mooy B. A. S. (2016). LOBSTAHS: An Adduct-Based Lipidomics Strategy for Discovery and Identification of Oxidative Stress Biomarkers. *Anal. Chem.* 88 7154–7162. 10.1021/acs.analchem.6b01260 27322848

[B18] Coolen M. J. L. Abbas B. van Bleijswijk J. Hopmans E. C. Kuypers M. M. M. Wakeham S. G. (2007). Putative ammonia-oxidizing Crenarchaeota in suboxic waters of the Black Sea: a basin-wide ecological study using 16S ribosomal and functional genes and membrane lipids. *Environ. Microbiol.* 9 1001–1016. 10.1111/j.1462-2920.2006.01227.x 17359272

[B19] Cutignano A. Luongo E. Nuzzo G. Pagano D. Manzo E. Sardo A. (2016). Profiling of complex lipids in marine microalgae by UHPLC/tandem mass spectrometry. *Algal Res.* 17 348–358. 10.1016/j.algal.2016.05.016

[B20] Cvejic J. H. Bodrossy L. Kovács K. L. Rohmer M. (2000). Bacterial triterpenoids of the hopane series from the methanotrophic bacteria Methylocaldum spp.: Phylogenetic implications and first evidence for an unsaturated aminobacteriohopanepolyol. *FEMS Microbiol. Lett.* 182 361–365. 10.1016/S0378-1097(99)00610-2 10620693

[B21] Dembitsky V. M. (1996). Betaine ether-linked glycerolipids: chemistry and biology. *Prog. Lipid Res.* 35 1–51. 10.1016/0163-7827(95)00009-7 9039425

[B22] Ding S. Bale N. J. Hopmans E. C. Villanueva L. Arts M. Schouten S. (2021). Lipidomics of environmental microbial communities. II: Characterization using molecular networking and information theory. *Front. Microbiol.* 2021:659315. 10.3389/fmicb.2021.659315 PMC8311935 34322097

[B23] Ding S. Lange M. Lipp J. Schwab V. F. Chowdhury S. Pollierer M. M. (2020). Characteristics and origin of intact polar lipids in soil organic matter. *Soil Biol. Biochem.* 151:108045. 10.1016/j.soilbio.2020.108045

[B24] Elling F. J. Könneke M. Nicol G. W. Stieglmeier M. Bayer B. Spieck E. (2017). Chemotaxonomic characterisation of the thaumarchaeal lipidome. *Environ. Microbiol.* 19 2681–2700. 10.1111/1462-2920.13759 28419726

[B25] Ertefai T. F. Fisher M. C. Fredricks H. F. Lipp J. S. Pearson A. Birgel D. (2008). Vertical distribution of microbial lipids and functional genes in chemically distinct layers of a highly polluted meromictic lake. *Org. Geochem.* 39 1572–1588. 10.1016/j.orggeochem.2008.07.009

[B26] Gaskell S. J. Eglinton G. (1976). Sterols of a contemporary lacustrine sediment. *Geochim. Cosmochim. Acta* 40 1221–1228. 10.1016/0016-7037(76)90157-5

[B27] Gibson R. A. van der Meer M. T. J. Hopmans E. C. Reysenbach A.-L. Schouten S. Sinninghe Damsté J. S. (2013). Comparison of intact polar lipid with microbial community composition of vent deposits of the Rainbow and Lucky Strike hydrothermal fields. *Geobiology* 11 72–85. 10.1111/gbi.12017 23231657

[B28] Godchaux W. III. Leadbetter E. R. (1980). Capnocytophaga spp. contain sulfonolipids that are novel in procaryotes. *J. Bacteriol*. 144 592–602. 10.1128/jb.144.2.592-602.1980 6253439 PMC294706

[B29] Grossi V. Mollex D. Vinçon-Laugier A. Hakil F. Pacton M. Cravo-Laureau C. (2015). Mono- and Dialkyl Glycerol Ether Lipids in Anaerobic Bacteria: Biosynthetic Insights from the Mesophilic Sulfate Reducer Desulfatibacillum alkenivorans PF2803T. *Appl. Environ. Microbiol.* 81 3157–3168. 10.1128/AEM.03794-14 25724965 PMC4393425

[B30] Guschina I. A. Harwood J. L. (2009). “Algal lipids and effect of the environment on their biochemistry,” in *Lipids in Aquatic Ecosystems*, eds Kainz M. Brett M. T. Arts M. T. (New York, NY: Springer), 1–24. 10.1007/978-0-387-89366-2_1

[B31] Gutiérrez M. H. Vera J. Srain B. Quiñones R. A. Wörmer L. Hinrichs K.-U. (2020). Biochemical fingerprints of marine fungi: implications for trophic and biogeochemical studies. *Aquat. Microb. Ecol.* 84 75–90. 10.3354/ame01927

[B32] Harvey H. R. Fallon R. D. Patton J. S. (1986). The effect of organic matter and oxygen on the degradation of bacterial membrane lipids in marine sediments. *Geochim. Cosmochim. Acta* 50 795–804. 10.1016/0016-7037(86)90355-8

[B33] Hay B. J. Honjo S. Kempe S. Ittekkot V. A. Degens E. T. Konuk T. (1990). Interannual variability in particle flux in the southwestern Black Sea. *Deep Sea Res. Part A Oceanogr. Res. Papers* 37 911–928. 10.1016/0198-0149(90)90103-3

[B34] Heaver S. L. Johnson E. L. Ley R. E. (2018). Sphingolipids in host-microbial interactions. *Curr Opin Microbiol.* 43, 92–99. 10.1016/j.mib.2017.12.011 29328957

[B35] Hölzl G. Dörmann P. (2007). Structure and function of glycoglycerolipids in plants and bacteria. *Prog. Lipid Res.* 46 225–243. 10.1016/j.plipres.2007.05.001 17599463

[B36] Hopmans E. C. Smit N. Schwartz-Narbonne R. Sinninghe Damsté J. S. Rush D. (2021). Analysis of non-derivatized bacteriohopanepolyols using UHPLC-HRMS reveals great structural diversity in environmental lipid assemblages. *Organic Geochem.* [Preprint].

[B37] Hsu F.-F. Turk J. (1999). Structural characterization of triacylglycerols as lithiated adducts by electrospray ionization mass spectrometry using low-energy collisionally activated dissociation on a triple stage quadrupole instrument. *J. Am. Soc. Mass Spectromet.* 10 587–599. 10.1016/S1044-0305(99)00035-5 10384723

[B38] Hunter J. E. Frada M. J. Fredricks H. F. Vardi A. Van Mooy B. A. S. (2015). Targeted and untargeted lipidomics of Emiliania huxleyi viral infection and life cycle phases highlights molecular biomarkers of infection, susceptibility, and ploidy. *Front. Mar. Sci.* 2:00081. 10.3389/fmars.2015.00081

[B39] Kato M. Sakai M. Adachi K. Ikemoto H. Sano H. (1996). Distribution of betaine lipids in marine algae. *Phytochemistry* 42 1341–1345. 10.1016/0031-9422(96)00115-X

[B40] Koga Y. Morii H. Akagawa-Matsushita M. Ohga M. (1998). Correlation of Polar Lipid Composition with 16S rRNA Phylogeny in Methanogens. Further Analysis of Lipid Component Parts. *Biosci. Biotechnol. Biochem.* 62 230–236. 10.1271/bbb.62.230 27388514

[B41] Koopmans M. P. Köster J. Van Kaam-Peters H. M. E. Kenig F. Schouten S. Hartgers W. A. (1996). Diagenetic and catagenetic products of isorenieratene: Molecular indicators for photic zone anoxia. *Geochim. Cosmochim. Acta* 60 4467–4496. 10.1016/S0016-7037(96)00238-4

[B42] Kraal P. Dijkstra N. Behrends T. Slomp C. P. (2017). Phosphorus burial in sediments of the sulfidic deep Black Sea: Key roles for adsorption by calcium carbonate and apatite authigenesis. *Geochim. Cosmochim. Acta* 204 140–158. 10.1016/j.gca.2017.01.042

[B43] Kuypers M. M. M. Sliekers A. O. Lavik G. Schmid M. Jørgensen B. B. Kuenen J. G. (2003). Anaerobic ammonium oxidation by anammox bacteria in the Black Sea. *Nature* 422 608–611. 10.1038/nature01472 12686999

[B44] Li X. He Q. Hou H. Zhang S. Zhang X. Zhang Y. (2018). Targeted lipidomics profiling of marine phospholipids from different resources by UPLC-Q-Exactive Orbitrap/MS approach. *J. Chromatogr. B* 1096 107–112. 10.1016/j.jchromb.2018.08.018 30165287

[B45] Li Y. Lou Y. Mu T. Xu J. Zhou C. Yan X. (2017). Simultaneous structural identification of diacylglyceryl-N-trimethylhomoserine (DGTS) and diacylglycerylhydroxymethyl-N,N,N-trimethyl-β-alanine (DGTA) in microalgae using dual Li+/H+ adduct ion mode by ultra-performance liquid chromatography/quadrupole time-of-flight mass spectrometry. *Rapid Communicat. Mass Spectrom.* 31 457–468. 10.1002/rcm.7818 28040883

[B46] Liebisch G. Vizcaíno J. A. Köfeler H. Trötzmüller M. Griffiths W. J. Schmitz G. (2013). Shorthand notation for lipid structures derived from mass spectrometry. *J. Lipid Res.* 54 1523–1530. 10.1194/jlr.M033506 23549332 PMC3646453

[B47] Lipp J. S. Hinrichs K.-U. (2009). Structural diversity and fate of intact polar lipids in marine sediments. *Geochim. Cosmochim. Acta* 73 6816–6833. 10.1016/j.gca.2009.08.003

[B48] Liu X.-L. Lipp J. S. Simpson J. H. Lin Y.-S. Summons R. E. Hinrichs K.-U. (2012a). Mono- and dihydroxyl glycerol dibiphytanyl glycerol tetraethers in marine sediments: Identification of both core and intact polar lipid forms. *Geochim. Cosmochim. Acta* 89 102–115. 10.1016/j.gca.2012.04.053

[B49] Liu X.-L. Summons R. E. Hinrichs K.-U. (2012b). Extending the known range of glycerol ether lipids in the environment: structural assignments based on tandem mass spectral fragmentation patterns. *Rapid Communicat. Mass Spectromet.* 26 2295–2302. 10.1002/rcm.6355 22956321

[B50] Longo W. M. Huang Y. Yao Y. Zhao J. Giblin A. E. Wang X. (2018). Widespread occurrence of distinct alkenones from Group I haptophytes in freshwater lakes: Implications for paleotemperature and paleoenvironmental reconstructions. *Earth Planet. Sci. Lett.* 492 239–250. 10.1016/j.epsl.2018.04.002

[B51] Louda J. W. Li J. Liu L. Winfree M. N. Baker E. W. (1998). Chlorophyll-a degradation during cellular senescence and death. *Org. Geochem.* 29 1233–1251. 10.1016/S0146-6380(98)00186-7

[B52] Louda J. W. Liu L. Baker E. W. (2002). Senescence- and death-related alteration of chlorophylls and carotenoids in marine phytoplankton. *Org. Geochem.* 33 1635–1653. 10.1016/s0146-6380(02)00106-7

[B53] Moncheva S. Gotsis-Skretas O. Pagou K. Krastev A. (2001). Phytoplankton Blooms in Black Sea and Mediterranean Coastal Ecosystems Subjected to Anthropogenic Eutrophication: Similarities and Differences. *Estuar. Coastal Shelf Sci.* 53 281–295. 10.1006/ecss.2001.0767

[B54] Moore E. K. Hopmans E. C. Rijpstra W. I. C. Villanueva L. Dedysh S. N. Kulichevskaya I. S. (2013). Novel Mono-, Di-, and Trimethylornithine Membrane Lipids in Northern Wetland Planctomycetes. *Appl. Environ. Microbiol.* 79 6874–6884. 10.1128/AEM.02169-13 23995937 PMC3811537

[B55] Moore E. K. Hopmans E. C. Rijpstra W. I. C. Villanueva L. Sinninghe Damsté J. S. (2016). Elucidation and identification of amino acid containing membrane lipids using liquid chromatography/high-resolution mass spectrometry: LC/HRMS of amino acid containing membrane lipids. *Rapid Communicat. Mass Spectromet.* 30 739–750. 10.1002/rcm.7503 27281845

[B56] Murray J. W. Lee B.-S. Bullister J. Luther G. W. (1999). “The Suboxic Zone of the Black Sea,” in *Environmental Degradation of the Black Sea: Challenges and Remedies* NATO Science Series, eds Beşiktepe S. T. Ünlüata Ü Bologa A. S. (Dordrecht: Springer Netherlands), 75–91. 10.1007/978-94-011-4568-8_6

[B57] Neretin L. N. Abed R. M. M. Schippers A. Schubert C. J. Kohls K. Kuypers M. M. M. (2007). Inorganic carbon fixation by sulfate-reducing bacteria in the Black Sea water column. *Environ. Microbiol.* 9 3019–3024. 10.1111/j.1462-2920.2007.01413.x 17991030

[B58] Overmann J. Cypionka H. Pfennig N. (1992). An extremely low−light adapted phototrophic sulfur bacterium from the Black Sea. *Limnol. Oceanogr.* 37 150–155. 10.4319/LO.1992.37.1.0150

[B59] Pitcher A. Hopmans E. C. Mosier A. C. Park S.-J. Rhee S.-K. Francis C. A. (2011). Core and intact polar glycerol dibiphytanyl glycerol tetraether lipids of ammonia-oxidizing archaea enriched from marine and estuarine sediments. *Appl. Environ. Microbiol.* 77 3468–3477. 10.1128/AEM.02758-10 21441324 PMC3126447

[B60] Pluskal T. Castillo S. Villar-Briones A. Orešiè M. (2010). MZmine 2: Modular framework for processing, visualizing, and analyzing mass spectrometry-based molecular profile data. *BMC Bioinformatics* 11:395. 10.1186/1471-2105-11-395 20650010 PMC2918584

[B61] Pluskal T. Korf A. Smirnov A. Schmid R. Fallon T. R. Du X. (2020). “CHAPTER 7:Metabolomics Data Analysis Using MZmine,” in *Processing Metabolomics and Proteomics Data with Open Software*, ed. Winkler R. (London: ıRoyal Society of Chemistry).

[B62] Popendorf K. J. Lomas M. W. Van Mooy B. A. S. (2011). Microbial sources of intact polar diacylglycerolipids in the Western North Atlantic Ocean. *Org. Geochem.* 42 803–811. 10.1016/j.orggeochem.2011.05.003

[B63] Qin W. Carlson L. T. Armbrust E. V. Devol A. H. Moffett J. W. Stahl D. A. (2015). Confounding effects of oxygen and temperature on the TEX86 signature of marine Thaumarchaeota. *PNAS* 112 10979–10984. 10.1073/pnas.1501568112 26283385 PMC4568219

[B64] Repeta D. J. (1993). A high resolution historical record of Holocene anoxygenic primary production in the Black Sea. *Geochim. Cosmochim. Acta* 57 4337–4342. 10.1016/0016-7037(93)90334-S

[B65] Repeta D. J. Simpson D. J. (1991). The distribution and recycling of chlorophyll, bacteriochlorophyll and carotenoids in the Black Sea. *Deep Sea Res. Part A Oceanogr. Res. Papers* 38 S969–S984. 10.1016/S0198-0149(10)80019-6

[B66] Repeta D. J. Simpson D. J. Jorgensen B. B. Jannasch H. W. (1989). Evidence for anoxygenic photosynthesis from the distribution of bacteriochlorophylls in the Black Sea. *Nature* 342 69–72. 10.1038/342069a0 11536615

[B67] Rossel P. E. Lipp J. S. Fredricks H. F. Arnds J. Boetius A. Elvert M. (2008). Intact polar lipids of anaerobic methanotrophic archaea and associated bacteria. *Organic Geochem.* 39 992–999. 10.1016/j.orggeochem.2008.02.021

[B68] Rush D. Sinninghe Damsté J. S. (2017). Lipids as paleomarkers to constrain the marine nitrogen cycle. *Environ. Microbiol.* 19 2119–2132. 10.1111/1462-2920.13682 28142226 PMC5516240

[B69] Rush D. Osborne K. A. Birgel D. Kappler A. Hirayama H. Peckmann J. (2016). The Bacteriohopanepolyol Inventory of Novel Aerobic Methane Oxidising Bacteria Reveals New Biomarker Signatures of Aerobic Methanotrophy in Marine Systems. *PLoS One* 11:e0165635. 10.1371/journal.pone.0165635 27824887 PMC5100885

[B70] Saleem A. Bell M. A. Kimpe L. E. Korosi J. B. Arnason J. T. Blais J. M. (2019). Identifying novel treeline biomarkers in lake sediments using an untargeted screening approach. *Sci. Tot. Environ.* 694:133684. 10.1016/j.scitotenv.2019.133684 31398651

[B71] Schouten S. Hopmans E. C. Sinninghe Damsté J. S. (2013). The organic geochemistry of glycerol dialkyl glycerol tetraether lipids: A review. *Org. Geochem.* 54 19–61. 10.1016/j.orggeochem.2012.09.006

[B72] Schouten S. Hopmans E. C. Baas M. Boumann H. Standfest S. Koenneke M. (2008). Intact membrane lipids of “*Candidatus* Nitrosopumilus maritimus,” a cultivated representative of the cosmopolitan mesophilic group I crenarchaeota. *Appl. Environ. Microbiol.* 74 2433–2440. 10.1128/AEM.01709-07 18296531 PMC2293165

[B73] Schubotz F. Wakeham S. G. Lipp J. S. Fredricks H. F. Hinrichs K.-U. (2009). Detection of microbial biomass by intact polar membrane lipid analysis in the water column and surface sediments of the Black Sea. *Environ. Microbiol.* 11 2720–2734. 10.1111/j.1462-2920.2009.01999.x 19624710

[B74] Schubotz F. Xie S. Lipp J. S. Hinrichs K.-U. Wakeham S. G. (2018). Intact polar lipids in the water column of the eastern tropical North Pacific: abundance and structural variety of non-phosphorus lipids. *Biogeosciences* 15 6481–6501. 10.5194/bg-15-6481-2018

[B75] Sinninghe Damsté J. S. Rijpstra W. I. C. Hopmans E. C. Prahl F. G. Wakeham S. G. Schouten S. (2002a). Distribution of Membrane Lipids of Planktonic Crenarchaeota in the Arabian Sea. *Appl. Environ. Microbiol.* 68 2997–3002. 10.1128/AEM.68.6.2997-3002.2002 12039760 PMC123986

[B76] Sinninghe Damsté J. S. Schouten S. van Duin A. C. T. (2001). Isorenieratene derivatives in sediments: possible controls on their distribution. *Geochim. Cosmochim. Acta* 65 1557–1571. 10.1016/S0016-7037(01)00549-X

[B77] Sinninghe Damsté J. S. Schouten S. Hopmans E. C. van Duin A. C. T. Geenevasen J. A. J. (2002b). Crenarchaeol: the characteristic core glycerol dibiphytanyl glycerol tetraether membrane lipid of cosmopolitan pelagic crenarchaeota. *J. Lipid Res.* 43 1641–1651. 12364548 10.1194/jlr.m200148-jlr200

[B78] Sollai M. Villanueva L. Hopmans E. C. Reichart G.-J. Sinninghe Damsté J. S. (2018). A combined lipidomic and 16S rRNA gene amplicon sequencing approach reveals archaeal sources of intact polar lipids in the stratified Black Sea water column. *Geobiology* 17 91–109. 10.1111/gbi.12316 30281902 PMC6586073

[B79] Sturt H. F. Summons R. E. Smith K. Elvert M. Hinrichs K. U. (2004). Intact polar membrane lipids in prokaryotes and sediments deciphered by high-performance liquid chromatography/electrospray ionization multistage mass spectrometry - new biomarkers for biogeochemistry and microbial ecology. *Rapid Commun. Mass Spectrom.* 18 617–628. 10.1002/rcm.1378 15052572

[B80] Summons R. E. Thomas J. Maxwell J. R. Boreham C. J. (1992). Secular and environmental constraints on the occurrence of dinosterane in sediments. *Geochim. Cosmochim. Acta* 56 2437–2444. 10.1016/0016-7037(92)90200-3

[B81] Talbot H. M. Handley L. Spencer-Jones C. L. Bienvenu D. J. Schefuß E. Mann P. J. (2014). Variability in aerobic methane oxidation over the past 1.2 Myrs recorded in microbial biomarker signatures from Congo fan sediments. *Geochim. Cosmochim. Acta* 133 387–401. 10.1016/j.gca.2014.02.035

[B82] Van Mooy B. A. S. Fredricks H. F. (2010). Bacterial and eukaryotic intact polar lipids in the eastern subtropical South Pacific: Water-column distribution, planktonic sources, and fatty acid composition. *Geochim. Cosmochim. Acta* 74 6499–6516. 10.1016/j.gca.2010.08.026

[B83] Villanueva L. Damsté J. S. S. Schouten S. (2014). A re-evaluation of the archaeal membrane lipid biosynthetic pathway. *Nat. Rev. Microbiol.* 12 438–448. 10.1038/nrmicro3260 24801941

[B84] Wakeham S. G. (1985). Wax esters and triacylglycerols in sinking particulate matter in the Peru upwelling area (15°S, 75°W). *Mar. Chem.* 17 213–235. 10.1016/0304-4203(85)90012-X

[B85] Wakeham S. G. Amann R. Freeman K. H. Hopmans E. C. Jørgensen B. B. Putnam I. F. (2007). Microbial ecology of the stratified water column of the Black Sea as revealed by a comprehensive biomarker study. *Organic Geochem.* 38 2070–2097. 10.1016/j.orggeochem.2007.08.003

[B86] Wakeham S. G. Lewis C. M. Hopmans E. C. Schouten S. Sinninghe Damsté J. S. (2003). Archaea mediate anaerobic oxidation of methane in deep euxinic waters of the Black Sea. *Geochim. Cosmochim. Acta* 67 1359–1374. 10.1016/S0016-7037(02)01220-6

[B87] Ward J. H. (1963). Hierarchical Grouping to Optimize an Objective Function. *J. Am. Statist. Associat.* 58 236–244. 10.1080/01621459.1963.10500845

[B88] White D. C. Davis W. M. Nickels J. S. King J. D. Bobbie R. J. (1979). Determination of the sedimentary microbial biomass by extractible lipid phosphate. *Oecologia* 40 51–62. 10.1007/BF00388810 28309603

[B89] Wörmer L. Lipp J. S. Schröder J. M. Hinrichs K.-U. (2013). Application of two new LC–ESI–MS methods for improved detection of intact polar lipids (IPLs) in environmental samples. *Organic Geochem.* 59 10–21. 10.1016/j.orggeochem.2013.03.004

[B90] Yadav S. Villanueva L. Bale N. Koenen M. Hopmans E. C. Damsté J. S. S. (2020). Physiological, chemotaxonomic and genomic characterization of two novel piezotolerant bacteria of the family Marinifilaceae isolated from sulfidic waters of the Black Sea. *Systemat. Appl. Microbiol.* 43:126122. 10.1016/j.syapm.2020.126122 32847788

[B91] Yoshinaga M. Y. Lazar C. S. Elvert M. Lin Y.-S. Zhu C. Heuer V. B. (2015). Possible roles of uncultured archaea in carbon cycling in methane-seep sediments. *Geochim. Cosmochim. Acta* 164 35–52. 10.1016/j.gca.2015.05.003

[B92] Zeng J. Liu S. Cai W. Jiang H. Lu X. Li G. (2019). Emerging lipidome patterns associated with marine Emiliania huxleyi-virus model system. *Sci. Tot. Environ.* 688 521–528. 10.1016/j.scitotenv.2019.06.284 31254817

[B93] Zhu C. Meador T. B. Dummann W. Hinrichs K.-U. (2014a). Identification of unusual butanetriol dialkyl glycerol tetraether and pentanetriol dialkyl glycerol tetraether lipids in marine sediments. *Rapid Communicat. Mass Spectromet.* 28 332–338. 10.1002/rcm.6792 24395500

[B94] Zhu C. Yoshinaga M. Y. Peters C. A. Liu X.-L. Elvert M. Hinrichs K.-U. (2014b). Identification and significance of unsaturated archaeal tetraether lipids in marine sediments. *Rapid Communicat. Mass Spectromet.* 28 1144–1152. 10.1002/rcm.6887 24711277

