# Supplementary material for: Lipidomics of Environmental Microbial Communities. I: Visualization of Component Distributions Using Untargeted Analysis of High-Resolution Mass Spectrometry Data
Source: Front Microbiol. 2021 Jul 23;12:659302. doi: 10.3389/fmicb.2021.659302 (PMC8343106; doi:10.3389/fmicb.2021.659302)
Supplement: Supplementary file 3 [file Table_1.DOCX]

**Table S1. LCMS contaminants observed.**

| ***m/z* of component** | **AEC** | **Δ (mmu)** | **MS/MS fragments** | **Assignment** |
| --- | --- | --- | --- | --- |
| 684.2025 | C_18_H_58_Si_9_O_9_N | 0.4 | 359.0275, 429.0876 | Polysiloxane |
| 758.2211 | C_20_H_64_Si_10_O_10_N | 0.6 | 299.0609, 359.0275 | Polysiloxane |
| 832.2397 | C_22_H_70_Si_11_O_11_N | 0.8 | 299.0609, 355.0692 | Polysiloxane |
| 675.6761 | C_44_H_87_O_2_N_2_ | 0.1 | 338.3409 | Erucamide |
| 610.1844 | C_16_H_52_Si_8_O_8_N | 0.2 | 355.0695 | Polysiloxane |
| 672.1998 |  |  | 355.0696 | Polysiloxane |
| 536.1651 | C_14_H_46_Si_7_O_7_N | 0.3 | 299.0612 | Polysiloxane |
| 537.5359 | C_34_H_69_O_2_N_2_ | 0.5 | 282.2788 | Diamide |
